# Neuron-to-neuron α-synuclein propagation *in vivo* is independent of neuronal injury

**DOI:** 10.1186/s40478-015-0198-y

**Published:** 2015-03-24

**Authors:** Ayse Ulusoy, Ruth E Musgrove, Raffaella Rusconi, Michael Klinkenberg, Michael Helwig, Anja Schneider, Donato A Di Monte

**Affiliations:** German Center for Neurodegenerative Diseases (DZNE), Ludwig-Erhard-Allee 2, 53175 Bonn, Germany; German Center for Neurodegenerative Diseases (DZNE), Göttingen, Germany; Department of Psychiatry and Psychotherapy, University Medicine Göttingen, Göttingen, Germany

**Keywords:** Adeno-associated viral vector, Neurodegeneration, Parkinson’s disease, Rat, Vagus nerve

## Abstract

**Introduction:**

Interneuronal propagation of α-synuclein has been demonstrated in a variety of experimental models and may be involved in disease progression during the course of human synucleinopathies. The aim of this study was to assess the role that neuronal injury or, *vice versa*, cell integrity could have in facilitating interneuronal α-synuclein transfer and consequent protein spreading in an *in vivo* animal model.

**Results:**

Viral vectors carrying the DNA for human α-synuclein were injected into the rat vagus nerve to trigger protein overexpression in the medulla oblongata and consequent spreading of human α-synuclein toward pons, midbrain and forebrain. Two vector preparations sharing the same viral construct were manufactured using identical procedures with the exception of methods for their purification. They were also injected at concentrations that induced comparable levels of α-synuclein transduction/overexpression in the medulla oblongata. α-Synuclein load was associated with damage (at 6 weeks post injection) and death (at 12 weeks) of medullary neurons after treatment with only one of the two vector preparations. Of note, neuronal injury and degeneration was accompanied by a substantial reduction of caudo-rostral propagation of human α-synuclein.

**Conclusions:**

Interneuronal α-synuclein transfer, which underlies protein spreading from the medulla oblongata to more rostral brain regions in this rat model, is not a mere consequence of passive release from damaged or dead neurons. Neuronal injury and degeneration did not exacerbate α-synuclein propagation. In fact, data suggest that cell-to-cell passage of α-synuclein may be particularly efficient between intact, relatively healthy neurons.

**Electronic supplementary material:**

The online version of this article (doi:10.1186/s40478-015-0198-y) contains supplementary material, which is available to authorized users.

## Introduction

Neuron-to-neuron propagation of pathological forms of α-synuclein (α-syn) is a phenomenon of likely relevance for the development and progression of human α-synucleinopathies such as Parkinson’s disease (PD). Several lines of evidence support this assertion. Observations in post-mortem PD brain reveal a stereotypical accumulation of α-syn-containing Lewy bodies and Lewy neurites. Compatible with a mechanism of interneuronal α-syn spreading, these pathological lesions progressively affect interconnected brain regions, starting from the caudal brainstem and moving toward mesocortical and neocortical areas [1,2]. From the experimental standpoint, neuron-to-neuron α-syn transmission has been demonstrated *in vitro* in a variety of cell culture systems as well as *in vivo* in animal models [3-10]. Results of *in vivo* studies also support a relationship between α-syn propagation and neurodegenerative processes. When mice were injected intrastriatally with fibrillar α-syn, protein spreading not only reached neuronal populations distant from the injection site but also caused dopaminergic cell death in the substantia nigra [11]. In a separate model mimicking the spreading pattern of PD, interneuronal transmission of α-syn could be initiated by its overexpression in the rat medulla oblongata (MO); caudo-rostral spreading toward pons, midbrain and forebrain was accompanied by accumulation and aggregation of α-syn into swollen dystrophic axons [12].

Elucidation of the mechanisms involved in the transfer of α-syn (and/or pathological forms of it) from donor to recipient cells bears significant pathogenetic implications and could provide clues for therapeutic intervention targeting protein spreading. Experimental evidence suggests that α-syn could initially be secreted *via* membrane-bound vesicles, such as exosomes, and then taken up *via* endocytotic pathways, such as adsorptive endocytosis and dynamin-dependent endocytosis [3,4,13-16]. While these mechanisms would involve intact healthy cells, a critical unaddressed question remains the role that neuronal injury/death may play in facilitating α-syn access into the extracellular space. Passive release of α-syn from damaged neurons would be of particular relevance during neurodegenerative processes. Indeed, a vicious cycle could be envisioned by which pathological α-syn accumulation causes neuronal damage, neurodegeneration results in α-syn release, and extracellular α-syn becomes available for internalization into nearby neurons. Injury of these neurons would ultimately perpetuate the cycle and cause further propagation of α-syn pathology.

Experiments in this study were designed to determine the role of neuronal damage or, *vice versa*, cell integrity in α-syn propagation *in vivo*. α-Syn spreading from the caudal brainstem toward more rostral brain regions was triggered by protein overexpression in the rat MO and compared under experimental conditions characterized by absence of neurodegeneration *vs.* a loss of α-syn-containing neurons. Results demonstrated that passive release from injured neurons is not essential for triggering α-syn transmission, nor does it exacerbate protein spreading. In fact, α-syn propagation was more pronounced in the absence than in the presence of neurodegeneration, underscoring the importance of neuron-to-neuron α-syn transfer between intact, relatively healthy cells.

## Materials and methods

### Vectors

Recombinant adeno-associated virus (serotype 2 genome and serotype 6 capsid, AAV) was used for transgene expression of human wild-type α-synuclein (hα-syn) or enhanced green fluorescent protein (GFP) under the control of the human Synapsin1 promoter. Gene expression was enhanced using a woodchuck hepatitis virus post-transcriptional regulatory element (WPRE) and a polyadenylation signal sequence (polyA) [12,17]. Experiments compared the effects of two AAV preparations: AAV prep 1 (Vector Biolabs, Philadelphia, PA, USA) and AAV prep 2 (Sirion Biotech, Martinsried, Germany). For both preparations, 293 HEK cells were transfected with the same reporter plasmid (Additional file 1: Figure S1). Crude cell lysates containing the viral particles were then purified by either (i) two consecutive CsCl gradient centrifugations (AAV prep 1), or (ii) centrifugation through a discontinuous iodixanol gradient followed by heparin affinity chromatography (AAV prep 2). AAV preparations were concentrated and resuspended in phosphate buffered saline. Titration of the concentrated vectors was performed using quantitative PCR with primers against WPRE. Injected titers were 1 × 10^13^ genome copies/ml for hα-syn-AAV prep 1 and between 5 × 10^12^ and 1 × 10^13^ genome copies/ml for hα-syn-AAV prep 2. In experiments in which the effects of GFP overexpression were compared, GFP-AAV prep 1 or GFP-AAV prep 2 were injected at a titer of 1 ×10^13^ genome copies/ml.

### Animals and surgical procedure

Young adult female Sprague Dawley rats weighing 200–250 g were obtained from Charles River (Kisslegg, Germany). They were housed under a 12-h light/12-h dark cycle with free access to food and water. Experimental design and procedures were approved by the ethical committee of the State Agency for Nature, Environment and Consumer Protection in North Rhine Westphalia. Following anesthetization with 2% isoflurane mixed with O_2_ and N_2_O, a 2 cm-incision was made at the midline of the rat neck. The left vagus nerve was isolated from the surrounding tissue, and the vector solution (2 μl) was injected at a flow rate of 0.5 μl/min using a glass capillary (tip diameter = 60 μm) fitted onto a 5 μl Hamilton syringe. Control animals were injected with vehicle using the same volume and at the same flow rate. After injection, the capillary was kept in place for 3–4 minutes.

### Tissue preparation

Animals were killed under pentobarbital anesthesia and perfused through the ascending aorta with saline, followed by ice-cold 4% (w/v) paraformaldehyde. Brains were removed, immersion-fixed in 4% paraformaldehyde (for 24 h) and cryopreserved in 25% (w/v) sucrose solution. Coronal sections (40 μm) throughout the brain were cut using a freezing microtome and stored at −20°C in phosphate buffer (pH 7.4) containing 30% glycerol and 30% ethylene glycol.

### 3,3′-Diaminobenzidine staining

Free-floating brain sections were rinsed with Tris-buffered saline (TBS, pH 7.6). Endogenous peroxidase activity was quenched by incubation in a mixture of 3% H_2_O_2_ and 10% methanol in TBS. Non-specific binding sites were blocked using 5% normal serum in TBS containing 0.25% Triton-X-100 (TBS-T). Samples were then incubated overnight at room temperature in TBS-T containing 1% bovine serum albumin and primary antibodies: mouse anti-hα-syn clone syn211 (36–008, Merck Millipore, Darmstadt, Germany; 1:10,000 dilution), chicken anti-GFP (ab13970, Abcam; 1:10,000 dilution), and goat anti-choline acetyltransferase (AB144P, Millipore; 1:200 dilution). Sections were rinsed and incubated (for 1 h at room temperature) in biotinylated secondary antibody solution (horse anti-mouse BA2001 or rabbit anti-goat BA5000; Vector Laboratories, Burlingame, CA, USA; 1:200 dilution). Following treatment with avidin-biotin-peroxidase (ABC Elite kit, Vector Laboratories), color reaction was developed using 3,3′-diaminobenzidine kit (Vector Laboratories). Sections were mounted on coated slides, dried, stained (if appropriate) with cresyl violet (FD Neurotechnologies, Columbia, MD, USA) and coverslipped with Depex (Sigma-Aldrich, St. Louis, MO, USA).

Brightfield microscopy images were obtained using an IX2 UCB microscope from Olympus (Hamburg, Germany) equipped with a motorized stage (MBF Mac6000 System stage, MBF Biosciences). For z-stacking analysis, stacks were collected at 1 μm intervals, and a single image was generated by deep focus post-processing. Low magnification images of entire brain sections were generated using an Axioscan Z1 (Carl Zeiss, Göttingen, Germany) slide scanner.

### Fluorescence staining

Following blocking procedures (see above), tissue sections were incubated overnight with the following primary antibodies: mouse anti-hα-syn clone syn211 (36–008, Merck Millipore; 1:3,000 dilution), rabbit anti-poly ADP ribose polymerase (PARP) p85 fragment (G7341, Promega; 1:100 dilution), and mouse anti-hα-syn 5G4 (847–0102004001, Analytic Jena; 1:1000 dilution). Reactions with primary antibodies raised in mice were followed by incubations with biotinylated horse anti-mouse (BA2001, Vector Laboratories) secondary antibody. Streptavidin-conjugated DyLight fluorophore (DyLight 488, Vector Laboratories; 1:400 dilution) was then used for detection. Reactions with primary antibodies raised in rabbit were followed by incubations in the presence of DyLight 594-conjugated horse anti-rabbit secondary antibody (Vector Laboratories; 1:400 dilution). Sections were mounted on coated slides and coverslipped with PVA-DABCO (Sigma). Confocal fluorescence images were collected using a Zeiss LSM700 inverted laser scanning microscope.

### Histological quantifications

All histological evaluations were performed by investigators blinded to sample treatment. The total number of Nissl-positive and hα-syn-immunoreactive neurons was estimated in the dorsal motor nucleus of the vagus nerve (DMnX) using unbiased stereology [18]. Every sixth section throughout the entire DMnX was sampled. After delineation of the DMnX under a 10x objective (Additional file 2: Figure S2), counting was performed using a 60x Plan-Apo oil objective (Numerical aperture = 1.4). A guard zone thickness of 1 μm was set at the top and bottom of each section. Neurons were counted using the optical fractionator technique (Stereo Investigator software version 9, MBF Biosciences, Williston, VT, USA). The sampling interval in the X-Y coordinate axis was adjusted so that at least 100 cells were counted for each DMnX (sampling grid size: 125 × 125 mm; counting frame: 60 × 60 mm). Coefficient of error was calculated according to Gundersen and Jensen [19]; values <0.10 were accepted.

Every sixth section throughout the entire DMnX was used for volume estimates of DMnX neurons immunoreactive for hα-syn or choline acetyltransferase (ChAT). Measurements were made on (i) all hα-syn-labeled cells present in these sections, or (ii) a population of ChAT-positive neurons selected *via* stereological sampling. Volumes were calculated according to the isotropic nucleator method [20]. For each neuron, a nucleator probe was set to generate 6 random isotropic linear rays that emerged from a user-defined center point. The points at which the 6 rays touched the profile of the neuron were manually defined and used for volumetric estimates. Analyses were carried out using the Stereo Investigator software version 9 (MBF Biosciences).

Fluorescence intensity measurements were carried out on all α-syn-positive neurons present in three MO sections at the level of obex (Bregma: −13.8, −13.56 and −13.32 mm). For each neuron, confocal images were collected using a 60x objective at a single focal plane where the nucleus was clearly visible. All image settings including laser power, exposure time, gain, offset and scanning speed were kept constant throughout the measurements. For each neuron, background readings were obtained on empty areas at the same focal depth. Image analyses were carried out using the Fiji software (version 2.0) [21]. Corrected total cell fluorescence (CTCF) was calculated by the following formula: Integrated Density - (Area occupied by the selected cell x Mean fluorescence of background readings). CTCF values were averaged for each animal.

Quantification of hα-syn positive axons was made using sections at pre-defined Bregma coordinates [22]: −9.48 mm in pons, −7.80 mm in caudal midbrain, −6.00 mm in rostral midbrain and −2.40 mm in forebrain. At each section, all immunostained axons were counted using an Axioscope microscope (Carl Zeiss) under a 20x Plan-Apo objective.

The length of hα-syn-containing axons was estimated using the Space Balls stereological probe (Stereo Investigator software version 9, MBF Biosciences) [23]. Measurements were made on serial sections of the pons (Bregma: −9.7 to −9.22 mm) where an area encompassing the locus coeruleus and the parabrachialis nucleus was delineated. A virtual hemisphere (12 μm radius) was placed randomly within this area, and systematic sampling was performed at intervals of 100 μm in both X and Y axes. A guard zone of 1 μm was set at the top and bottom of each tissue section. While the microscope stage moved through the Z-axis, the circle diameter of the Space Balls hemisphere decreased concentrically at each 1 μm focal plane. The number of intersections between fibers and circles was counted and used to estimate a mean total fiber length. Volume of the reference region was estimated by the Cavalieri point-counting method applying a step length size of 200 μm [24]. Fiber density was calculated by the ratio total fiber length/volume of reference region.

### Reverse transcription PCR (RT-PCR)

Fixed tissue sections (40 μm) from rats injected with hα-syn-AAV preparations or vehicle were used for conventional or quantitative RT-PCR (qRT-PCR) analysis. Dorso-medial quadrants of the left (injected side) MO, which contained the DMnX, were dissected and pooled from equally spaced (2 sections every 160 μm) sections at Bregma −14.76 to −12.48 mm. Total RNA was extracted using the “RecoverAll Total Nucleic Acid Kit” (Ambion, Austin, TX, USA), and cDNA was synthesized by reverse transcription (SuperScript VILO Master Mix, Invitrogen, Carlsbad, CA, USA) using 100 ng of total RNA. The following primer sequences were used: (i) total (rat plus human) α-synuclein: 5'tggttttgtcaaaaaggaccag forward and 5'ccttcctcagaaggcatttc reverse; (ii) hypoxantine phosphoribosyltransferase 1 (housekeeping gene): 5'gaccggttctgtcatgtcg forward and 5'acctggttcatcatcactaatcac reverse, (iii) rat-only α-synuclein: 5'gagttctgcggaagcctagagagc forward and 5'gttttctcagcagcagccacaactcc reverse; and (iv) WPRE: 5'caattccgtggtgttgtcgg forward and 5'caaagggagatccgactcgt reverse. Analyses were performed in triplicates, using 1 μl of cDNA and Power SYBR Green Master Mix (Applied Biosystems Warrington, UK). For conventional RT-PCR the product was run on 1.5% agarose gel and visualized by EtBr staining. For qRT-PCR, measurements were performed using a StepOne plus real time PCR instrument with built-in software (Applied Biosystems). Relative quantities (fold changes) were calculated after normalization to housekeeping gene expression and calibration to a reference sample from vehicle-injected animals.

### Statistical analyses

Analyses were performed using JMP Pro Statistical software (version 10.0.0; SAS Institute, Cary, NC, USA). Means between two groups were compared with two-tailed *t*-test. Comparisons between multiple groups were carried out with one-way ANOVA followed by Tukey *post hoc* test. Statistical significance was set at *P* < 0.05.

## Results

### Viral vector-mediated α-syn transduction

**Figure 1:**
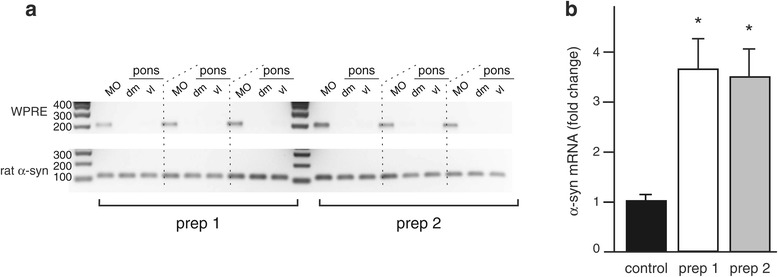
**Assessment of transduction efficiency at the mRNA level.** Analyses were performed at 6 weeks post treatment. **(a)** Rats received a single injection of hα-syn-AAV prep 1 (n = 3) or prep 2 (n = 3) into the vagus nerve. RT-PCR was performed to detect WPRE or, as a control, rat α-syn mRNA in DMnX-containing samples from the left (injected side) MO as well as in samples from the left dorso-medial (dm) and ventro-lateral (vl) pons. Specific bands were detected at 204 (WPRE) and 117 (rat a-syn) bp. **(b)** Animals received a single injection of vehicle (controls, n = 4), hα-syn-AAV prep 1 (n = 7) or hα-syn-AAV prep 2 (n = 7). Total (rat + human) α-syn mRNA levels were assayed by qRT-PCR in DMnX-containing samples from the left (injected side) MO. Values are means ± SEM. *P <0.05 *vs.* the control group (one-way ANOVA followed by Tukey’s post hoc test, F_2,17_ = 5.26).

Two separate preparations of hα-syn-carrying AAV, hα-syn-AAV prep 1 and hα-syn-AAV prep 2, were used for these experiments. Vector construct was identical for both preparations that only differed in regard to protocols employed for their purification (Additional file 1: Figure S1). Using an experimental paradigm previously described [12], AAV vectors were injected into the left vagus nerve in the rat neck. Animals were killed at 6 weeks post AAV treatment, and brain tissue was initially collected for RT-PCR measurements. To ascertain targeted transduction, amplification reactions evaluated the presence of WPRE, an enhancer element incorporated into the genome of the AAV vectors, in tissue specimens from the MO and pons. RT-PCR using WPRE-hybridizing primers confirmed hα-syn-AAV prep 1- and prep 2-mediated transfection in samples from the dorso-medial quadrant of the left (ipsilateral to vagal injections) MO containing the DMnX; in contrast, no WPRE mRNA was detected in pontine tissue (Figure 1a). Levels of transgene expression were then assessed by qRT-PCR measurements of total (rat plus human) α-syn mRNA in DMnX-containing specimens of the left MO. At the vector dilutions used for these studies, vagal injections with hα-syn-AAV prep 1 or prep 2 yielded samples with similar levels of α-syn overexpression. In these samples, total α-syn mRNA was increased by 3.7 (prep 1) and 3.6 (prep 2) folds (Figure 1b).

**Figure 2:**
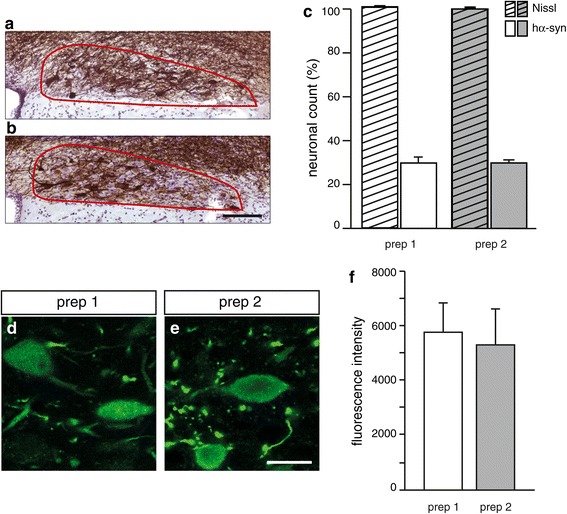
**Comparison of intraneuronal hα-syn expression after transduction with hα-syn-AAV prep 1** ***vs.*** **prep 2.** Analyses were performed at 6 weeks post treatment. **(a, b)** Representative sections of the MO (Bregma: −13.68 mm) were obtained from rats treated with **(a)** hα-syn-AAV prep 1 or **(b)** prep 2. Tissues were immunostained with anti-hα-syn and counterstained with cresyl violet. The DMnX is delineated in red. Scale bar in **(b)** = 100 μm. **(c)** Nissl-stained neurons (striped bars) and hα-syn-immunoreactive cells (solid bars) were counted in the left (injected side) DMnX of animals treated with hα-syn-AAV prep 1 (n = 7) or prep 2 (n = 7). Values (means ± SEM) are expressed as percent of the total number of Nissl-stained neurons. **(d, e)** Representative images of DMnX neurons immunostained with an anti-hα-syn antibody from rats treated with **(d)** hα-syn-AAV prep 1 or **(e)** prep 2. Scale bar in **(e)** = 20 μm. **(f)** Fluorescence intensity of hα-syn-immunoreactive DMnX neurons was measured by optical densitometry in rats treated with hα-syn-AAV prep 1 (n = 4) or prep 2 (n = 4). Values (pixels) are means ± SEM.

The extent of AAV-mediated transduction was also estimated histologically by calculating the percentages of hα-syn-containing neurons over the total number of neurons in the DMnX. Brains were collected postmortem at 6 weeks after vagal injections. The DMnX was delineated on tissue slices throughout the entire MO (Additional file 2: Figure S2), and stereological cell counting was performed after immunostaining with a specific hα-syn antibody and counterstaining with cresyl violet (Figure 2a and b). The percentage of transduced neurons was calculated by the formula: [number of cells that were both Nissl-stained and immunoreactive for hα-syn] / [total number of Nissl-positive neurons] x 100. Confirming previous results [12], no neurons immunoreactive for hα-syn were found in the right DMnX contralateral to the vagal injection side. In contrast, hα-syn-overexpressing cells were present in the left MO of AAV-treated rats and accounted for approximately 30% of the total DMnX neurons after injections with either hα-syn-AAV prep 1 or prep 2 (Figure 2c).

To further evaluate hα-syn transduction at the protein level, tissue sections of the left MO were stained for immunofluorescence with an anti-hα-syn antibody, and optical densitometry was used to measure the intensity of immunoreactivity within DMnX neurons. This semiquantitative analysis revealed no significant differences between samples from rats killed at 6 weeks after treatment with either hα-syn-AAV prep 1 or prep 2 (Figure 2d-f).

### Early pathological changes caused by AAV transduction

**Figure 3:**
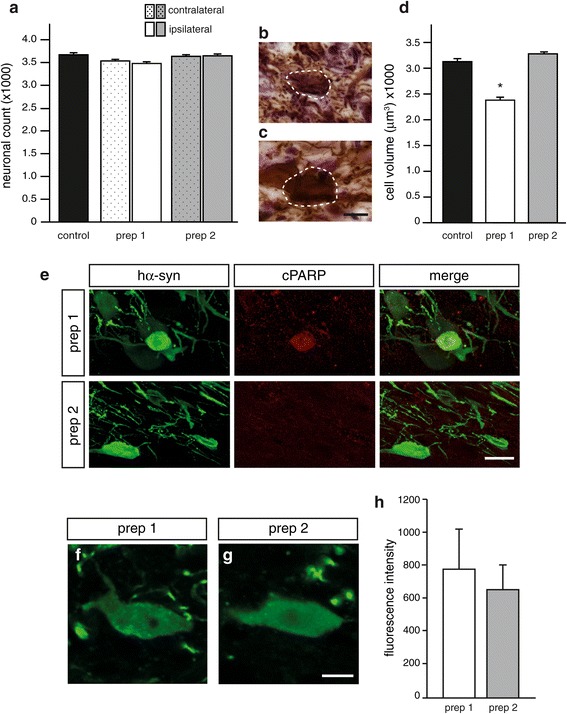
**Early (6 weeks post treatment) toxic effects of hα** **-syn-carrying AAV vectors. (a-d)** Rats received a single injection of vehicle (control animals, n = 3), hα-syn-AAV prep 1 (n = 7) or hα-syn-AAV prep 2 (n = 7) into the vagus nerve. **(a)** Stereological counts of Nissl-stained DMnX neurons were performed on tissue from the left (ipsilateral to AAV injections, solid bars) and right (contralateral to AAV injections, dotted bars) MO. Values are means ± SEM. **(b,c)** Representative sections of the MO (Bregma: −13.68 mm) were obtained from rats treated with **(b)** hα-syn-AAV prep 1 or **(c)** prep 2. Tissues were immunostained with an anti-hα-syn antibody. Images generated by z-stacking of two representative DMnX neurons are compared, showing an apparent decrease in cell size after transduction with hα-syn-AAV prep 1 **(b)**. Scale bar in **(c)** = 10 μm. **(d)** MO tissue sections were stained with either an antibody against ChAT (control tissue) or an anti-hα-syn antibody (rats injected with hα-syn-AAV prep 1 or prep 2). The volume (μm^3^) of ChAT-positive or hα-syn-immunoreactive neurons was estimated using a six-ray isotropic nucleator probe [20]. Measurements were made on 695 cells from control animals, 671 cells from prep 1-treated rats and 878 cells from prep 2-injected animals. For each group, single cell values were averaged. Values are means ± SEM. *P <0.0001 *vs.* the control or prep 2 groups (one-way ANOVA followed by Tukey’s post hoc test, F_2,2244_ = 51.15). **(e)** MO tissue sections from rats injected with hα-syn-AAV prep 1 or prep 2 were double-stained with antibodies against hα-syn and cleaved PARP (cPARP). Rare neurons (one of these neurons is shown in the upper panels) immunoreactive for both cPARP and hα-syn could be detected in hα-syn-AAV prep 1-treated animals. Scale bar = 20 μm. **(f-h)** DMnX-containing sections of the MO were immunostained with an antibody that preferentially reacts with aggregated forms of α-syn [25]. **(f,g)** Representative images of DMnX neurons from rats treated with **(f)** hα-syn-AAV prep 1 or **(g)** prep 2. Scale bar in **(g)** = 10 μm. **(h)** Fluorescence intensity of immunostained DMnX neurons was measured by optical densitometry in animals treated with hα-syn-AAV prep 1 (n = 4) or prep 2 (n = 4). Values (pixels) are means ± SEM.

Pathological changes were first evaluated in rats killed at 6 weeks post vagal injections. To determine whether AAV-mediated hα-syn transduction was associated with neurodegeneration, the number of Nissl-stained neurons was estimated by stereological cell counting in the DMnX of control untreated animals as well as in the right (contralateral to vagal injections) and left (ipsilateral) DMnX of AAV-injected rats. No cell death was observed as a consequence of transduction with either hα-syn-AAV prep 1 or prep 2. Indeed, the counts of DMnX neurons were not significantly different in control tissue as compared to samples from the right and left MO of AAV-treated animals (Figure 3a).

A second set of analyses assessed the possibility that, even in the absence of frank neurodegeneration, morphological (cell shrinkage) and/or biological (cleavage of PARP) signs of neuronal injury may be triggered by AAV transfection. Upon microscopic observation, the size of hα-syn-immunoreactive neurons appeared to be reduced in the DMnX of rats treated with hα-syn-AAV prep 1 as compared to hα-syn-AAV prep 2 (Figure 3b,c). Measurements using the stereological nucleator tool confirmed these observations; single cell volume was measured for several hundreds of unbiasedly selected neurons and averaged to obtain a mean cell volume. The mean volume of normal cholinergic DMnX neurons was estimated in tissue from control rats stained with an antibody against ChAT. No difference in cell volume was measured between these control neurons and neurons positive for hα-syn after transduction with hα-syn-AAV prep 2. Treatment with hα-syn-AAV prep 1 resulted instead in a >25% decrease in mean volume of hα-syn-overexpressing neurons (Figure 3d). Immunostaining with an antibody against the 85 kDa (p85) fragment of cleaved PARP (cPARP) was performed to assess caspase-dependent PARP cleavage as a marker of apoptotic processes. Rare neurons immunoreactive for both hα-syn and cPARP were seen in the left DMnX of rats injected with hα-syn-AAV prep 1, whereas no cPARP-positive cells could be detected in DMnX tissue from hα-syn-AAV prep 2-treated animals (Figure 3e).

Evidence of neuronal injury triggered by treatment with hα-syn-AAV prep 1 but not prep 2 prompted us to assess the possibility that differences in toxicity between the two preparations may be associated with changes in formation and accumulation of aggregated α-syn forms. Coronal sections of the left MO from prep 1- or prep 2-injected rats were immunostained with an antibody that has been shown to possess much higher reactivity toward aggregated as compared to monomeric α-syn [25]. While labeling was detected within DMnX neurons in all injected animals (Figure 3f and g), a comparison of fluorescence intensity by densitometric analysis did not reveal significant differences between the two treatment groups (Figure 3h).

### Later neurodegenerative changes induced by AAV transduction

**Figure 4:**
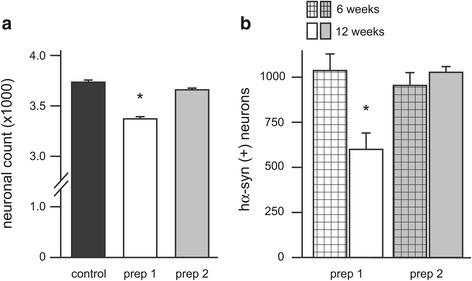
**Neurodegenerative changes caused by hα** **-syn-AAV prep 1 transduction. (a)** Rats received a single injection of vehicle (control animals, n = 3), hα-syn-AAV prep 1 (n = 5) or hα-syn-AAV prep 2 (n = 5) into the vagus nerve. Stereological counts of Nissl-stained DMnX neurons were performed at 12 weeks post treatment on tissue sections from the left (injected side) MO. Values are means ± SEM. *P <0.0001 *vs.* the control or prep 2 group (one-way ANOVA followed by Tukey’s post hoc test, F_2,12_ = 73.4). **(b)** The number of neurons immunoreactive for hα-syn was counted stereologically in the left (AAV-injected side) DMnX of rats injected with either hα-syn-AAV prep 1 or prep 2. Analyses were performed at 6 (bars with pattern, n = 7/group) or 12 weeks (solid bars, n = 5/group) post treatment. Values are means ± SEM. *P <0.005 *vs.* each of the other three groups (one-way ANOVA followed by Tukey’s post hoc test, F_3,23_ = 10.82).

To determine whether early cell injury at 6 weeks post transduction was ultimately followed by neurodegeneration, neuronal survival was evaluated in rats killed at 12 weeks after vagal injection. At this later time point, stereological cell count of DMnX neurons in the left MO revealed a reduction of total Nissl-stained cells of approximately 15% in animals injected with hα-syn-AAV prep 1; in contrast, no cell loss was triggered as a consequence of transduction with hα-syn-AAV prep 2 (Figure 4a). The number of DMnX neurons that were both Nissl-stained and immunoreactive for hα-syn was also significantly decreased at 12 weeks after AAV prep 1 treatment. This reduction was evident relative to (i) the number of hα-syn-transduced cells in rats injected with hα-syn-AAV prep 1 and killed at the 6-week time point, as well as (ii) the count of hα-syn-immunoreactive neurons in animals treated with hα-syn-AAV prep 2 and analyzed at either 6 or 12 weeks post transduction (Figure 4b).

The toxic properties of AAV prep 1 *vs.* prep 2 were further evaluated in a separate set of experiments in which rats received vagal injections of GFP- rather than hα-syn-carrying viral vectors. The number of Nissl-stained neurons was counted in the left DMnX at 12 weeks after treatment with either GFP-AAV prep 1 or GFP-AAV prep 2. Results showed a 10% reduction of cells in animals injected with GFP-AAV prep 1; no cell loss was instead observed after transduction with GFP-AAV prep 2 (Additional file 3: Figure S3). The decrease in neuronal count caused by treatment with GFP-AAV prep 1 was slightly less pronounced than the cell loss induced by hα-syn-AAV prep 1; this difference did not reach statistical significance, however.

### Propagation of hα-syn from the MO to pons

**Figure 5:**
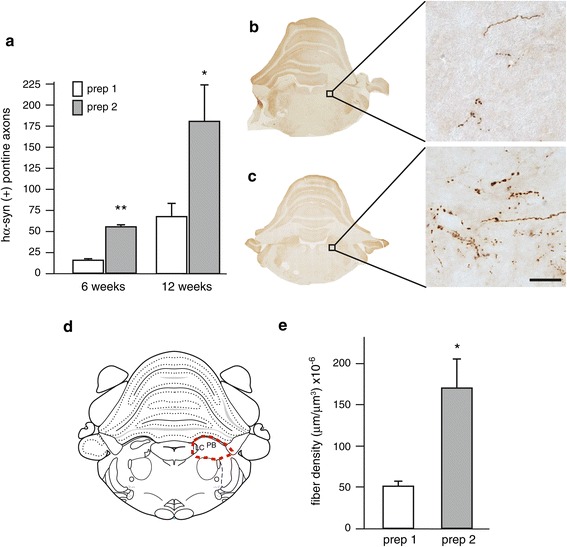
**Spreading of hα** **-syn to the pons after AAV-mediated transduction of MO neurons. (a)** Rats received a single injection of hα-syn-AAV prep 1 (open bars) or hα-syn-AAV prep 2 (filled bars) into the vagus nerve. The number of axonal projections immunostained with an anti-hα-syn antibody was counted in the left (injected side) pons at 6 (n = 7/group) and 12 (n = 5/group) weeks post injection. Values are means ± SEM. **P <0.0001 *vs.* the prep 1 group at 6 weeks (*t* test, F_1,13_ = 120.99). *P <0.05 *vs.* the prep 1 group at 12 weeks (*t* test, F_1,9_ = 6.01). **(b, c)** Representative pontine sections (Bregma: −9.48 mm) were obtained from rats injected with **(b)** hα-syn-AAV prep 1 or **(c)** prep 2 and sacrificed at 12 weeks post treatment. Higher magnification images from the coeruleus-subcoeruleus complex were obtained by z-stacking. They show axonal projections immunostained with an anti-hα-syn antibody. Scale bar = 30 μm. **(d, e)** Rats received a single injection of hα-syn-AAV prep 1 (n = 5) or prep 2 (n = 5) and were killed at 12 weeks post treatment. **(d)** An area encompassing the locus coeruleus (LC) and the parabrachialis nucleus (PB) was delineated on serial sections throughout the pons (a schematic representation of one of these sections at Bregma −9.6 mm is shown), and **(e)** the density of fibers immunoreactive for hα-syn was estimated using the Space Balls stereological tool [23]. Values are means ± SEM. *P <0.05 *vs.* the prep 1 group (*T* test: F_1,9_ = 10.96).

Enhanced expression of hα-syn in the rat MO has been shown to trigger its interneuronal propagation toward more rostral brain regions [12]. Protein spreading was first assessed in coronal sections of the pons where axonal projections immunoreactive for hα-syn were counted at 6 and 12 weeks post-treatment. At both time points, the number of axons containing the exogenous protein was significantly higher in animals transduced with hα-syn-AAV prep 2 as compared to hα-syn-AAV prep 1 (Figure 5a). At 12 weeks, analyses of tissue sections by microscopic examination revealed that, in rats transduced with hα-syn-AAV prep 1, spreading from the MO affected only sparse axons in specific pontine regions, such as the coeruleus–subcoeruleus complex (Figure 5b). In these same regions of animals treated with hα-syn-AAV prep 2, propagation of the exogenous protein was indicated by the presence of numerous immunoreactive axons (Figure 5c). Pathological fibers loaded with hα-syn appeared as tortuous threads with intensely labeled varicosities; the presence of these swellings often produced an image of punctuated immunoreactivity on single-plane microscopy.

To confirm unbiased neuritic quantifications, the mean total length and density of fibers immunoreactive for hα-syn were estimated using the Space Balls stereological tool [23]. Measurements were carried out in pontine sections at 12 weeks post AAV injections. Random and systematic sampling was performed on an anatomically defined area encompassing the locus coeruleus and the parabrachialis nucleus (Figure 5d); this area was chosen because it represents a preferential spreading site within the pons. Results of these measurements confirmed a significant difference in protein propagation between the two treatment groups. The mean total length of hα-syn-containing axons was 72,973 ± 7,243 mm and 251,597 ± 50,341 mm after transduction with hα-syn-AAV prep 1 and prep 2, respectively. Fiber density was also more than three times greater in pontine sections from prep 2-injected rats (Figure 5e).

### Caudo-rostral spreading of hα-syn beyond the pons

**Figure 6:**
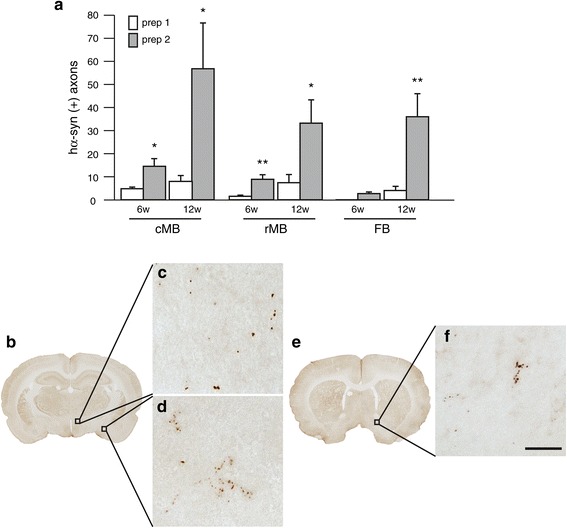
**Caudo-rostral spreading of hα-syn reaches midbrain and forebrain regions. (a)** Rats received a single injection of hα-syn-AAV prep 1 (open bars) or prep 2 (filled bars) into the vagus nerve. The number of axonal projections immunoreactive for hα-syn was counted in the left (injected side) caudal midbrain (cMB), rostral midbrain (rMB) and forebrain (FB) at 6 (n = 7/group) and 12 (n = 5/group) weeks post treatment. Values are means ± SEM. **P <0.01 and *P <0.05. *T* test: F_1,13_ = 7.88 and F_1,9_ = 5.88 in the cMB at 6 and 12 weeks, respectively; F_1,13_ = 10.10 and F_1,9_ = 5.86 in the rMB at 6 and 12 weeks, respectively; F_1,9_ = 9.94 in the FB at 12 weeks. **(b,e)** Representative sections of the forebrain at **(b)** Bregma: −2.40 mm and **(e)** Bregma: +0.48 mm were obtained from rats injected with AAV prep 2 and sacrificed at 12 weeks post treatment. **(c, d, f)** Higher magnification images from the **(c)** hypothalamus, **(d)** amygdala and **(f)** bed nucleus of the stria terminalis were generated by z-stacking. They show axonal projections immunostained with an anti-hα-syn antibody. Scale bar (for **c**, **d** and **f**) in **(f)** = 30 μm.

A final set of analyses involved counting the number of hα-syn-immunoreactive axons in coronal sections frontal to the pons. At 6 weeks, spreading of hα-syn triggered by AAV prep 1 reached the midbrain as its most rostral outpost. Progression of the spreading at 12 weeks was indicated by the occurrence of sparse fibers containing hα-syn in selected forebrain regions (e.g., hypothalamus and amygdala, Bregma: −2.40 mm) (Figure 6a). Treatment of rats with AAV prep 2 yielded counts of hα-syn-immunoreactive axons that were consistently higher than values seen with AAV prep 1 (Figure 6a). Labeled fibers were already present in forebrain sections at 6 weeks and became relatively abundant in the hypothalamus and amygdala at 12 weeks (Figure 6b-d). At this later time point following injections with AAV prep 2, a few axons were also detected in forebrain areas rostral to the anterior commissure (Bregma: +0.48 mm), such as the bed nucleus of the stria terminalis (Figure 6e and f).

## Discussion

Results of an earlier study demonstrated that propagation of hα-syn from the MO to more rostral regions of the rat brain could be triggered by AAV-mediated hα-syn transduction and was dependent on the levels of mRNA and protein overexpression. Very little spreading was observed when AAV transduction produced less than a one-fold increase in α-syn mRNA and a weak hα-syn immunostaining of MO neurons; this modest effect contrasted with the pronounced caudo-rostral propagation seen when mRNA levels were approximately three-fold higher than control values and labeling of histological MO sections revealed robust hα-syn immunoreactivity [12]. The purpose of this present investigation was to determine if other factors, besides intraneuronal expression levels, affected hα-syn spreading in this model. In particular, we aimed at assessing the relationship between neuronal injury and inter-neuronal α-syn propagation.

The two hα-syn-AAV preparations used in this study were injected into the rat vagus nerve at concentrations capable of inducing comparable levels of hα-syn overexpression in the MO. Despite this similarity, hα-syn-AAV prep 1 and prep 2 differed significantly with regard to their toxic effects, with neuronal damage being evident only after treatment with hα-syn-AAV prep 1. Contamination with impurities (e.g., empty capsids) and/or reagents used for their preparation (e.g., CsCl) is known to underlie the toxic potential of viral vectors [26-28]. Production of AAV prep 1 and prep 2 involved different purification procedures, which can result in various degrees of contamination. It is quite likely therefore that greater injury caused by hα-syn-AAV prep 1 arose from a relatively less thorough purification and a higher concentration of toxic byproducts.

Stereological cell counting of DMnX neurons revealed a reduction of total Nissl-stained cells at 12 weeks after treatment with hα-syn-AAV prep 1 as well as GFP-AAV prep 1. These data are consistent with non-specific toxic effects due to AAV prep 1 contamination. It is noteworthy that in rats injected with hα-syn-AAV prep 1 (i) the count of DMnX neurons immunoreactive for hα-syn was also decreased at the 12-week time point, and (ii) this decrease in hα-syn-positive cells (−437 ± 59 cells as compared to values at 6 weeks) fully accounted for the reduction in total Nissl-stained neurons (−438 ± 23 cells). Thus, despite the involvement of non-specific factors in AAV prep 1 toxicity, neuronal damage caused by hα-syn-AAV prep 1 still targeted DMnX neurons containing hα-syn. It is quite likely that protein overexpression made these cells particularly vulnerable to injury; if so, neurodegeneration might have arisen from the combined effects of hα-syn burden and AAV prep 1 toxicity.

Taken together, findings on neuronal transduction and cell damage in animals injected with hα-syn-AAV prep 1 *vs.* prep 2 revealed: (i) induction of comparable hα-syn overexpression, (ii) neuronal injury caused by one but not the other AAV preparation, and (iii) selectivity of this injury that targeted hα-syn-containing neurons. These features justified the following two assumptions concerning protein transmission after AAV prep 1- or prep 2-induced hα-syn overexpression. First, any potential difference in propagation would be unlikely to reflect changes in hα-syn levels at the site of initial protein transfer, i.e. MO neurons. Second, a comparison of spreading triggered by hα-syn-AAV prep 1 *vs.* prep 2 would be expected to yield different results should hα-syn transmission be significantly affected by damage/death of neurons.

Spreading was assessed by counting the number of axonal projections immunoreactive for hα-syn in brain regions rostral to the MO. Axons forming the rat vagus nerve originate from or terminate in the MO [29]; intravagal injections of hα-syn-carrying AAV vectors cause neuronal transduction and protein overexpression that are also confined to the MO [12]. Therefore, presence of hα-syn within axons that project from the pons, midbrain or forebrain to the MO [30-34] is indicative of caudo-rostral propagation *via* inter-neuronal protein transfer. Several lines of evidence from the present as well as earlier investigations support these assertions. Specific transduction is indicated by the pattern of AAV-induced overexpression that reproduces the anatomical brain distribution of efferent and afferent fibers forming the vagus nerve [12,29]. For example, because efferent vagal fibers originate from the DMnX and nucleus ambiguus [29], these two MO nuclei represent the only sites where, following AAV transduction, neuronal cell bodies immunoreactive for hα-syn are observed [12]. When markers of AAV transduction, such as hα-syn and WPRE mRNAs, were measured in the MO and pons, they could only be detected in specimens of the MO ipsilateral to vagal injections [12]. It would be highly unlikely for the serotype of AVV used in our experiments, i.e. AAV6, to be transferred intact across cells *via* mechanisms such as transcytosis [35]. Lack of cell bodies immunoreactive for hα-syn and lack of transcriptional AAV markers in brain regions rostral to the MO not only indicate specific transduction but also rule out the possibility that translocation of viral vectors rather than neuron-to-neuron transfer of hα-syn may underlie caudo-rostral spreading of the exogenous protein. Strong evidence supporting lack of transmission of viral particles also came from experiments in which rats received vagal injections of GFP- rather than hα-syn-AAV; in contrast to results in animals transduced with hα-syn, protein spreading was not observed in GFP-AAV-treated rats [12].

Overexpression of hα-syn caused by injections with either hα-syn-AAV prep 1 or prep 2 triggered caudo-rostral propagation as indicated by the finding that hα-syn-positive axons became progressively evident in the pons, midbrain and forebrain. Spreading after treatment with hα-syn-AAV prep 1 or prep 2 was consistently observed within areas such as the coeruleus–subcoeruleus complex (pons), dorsal raphae (midbrain) and amygdala (forebrain). Of note, the substantia nigra pars compacta (SNc) was not one of these predilection targets for hα-syn propagation. A likely explanation for these findings relates to anatomical brain connections since hα-syn-immunoreactive axons were primarily seen in regions with direct projections into the MO [30-34,36]. At the two time points considered in this study (6 and 12 weeks post treatment), spreading and accumulation of hα-syn beyond the MO affected neuritic projections while apparently sparing neuronal cell bodies; pathological changes were also evident in the form of enlarged dystrophic axons. Interestingly, a preferential axonal burden, as seen in this model of protein spreading, appears to be a feature common to a variety of experimental paradigms involving α-syn toxicity [9,37,38]. Greater vulnerability to neuritic accumulation/pathology could result, at least in part, from impaired degradation of axonal α-syn together with α-syn-induced disruption of axonal transport motor proteins [38,39]. Lack of cell body pathology and lack of hα-syn spreading to the SNc may be perceived as limitations of this experimental paradigm to reproduce PD features. On the other hand, findings are also consistent with the interpretation that this model of α-syn propagation triggered by protein overexpression in the MO mimics pathogenetic events that occur early in the disease process; early lesions may target axonal projections, involve a single trans-synaptic passage of α-syn and temporarily spare SNc neurons.

## Conclusions

Our present finding that spreading of hα-syn occurred after transduction with either hα-syn-AAV prep 1 or prep 2, i.e. in the presence or absence of neuronal injury/death, bears significant implications. Similarly, it is noteworthy that (i) the count of hα-syn-positive axons was much lower in the pons, midbrain and forebrain of rats injected with hα-syn-AAV prep 1 as compared to prep 2, and (ii) propagation of the exogenous protein toward regions rostral to the MO was less advanced when, as a consequence of hα-syn-AAV prep 1 administration, protein overexpression was associated with enhanced toxicity. Taken together, these results support the conclusion that release from damaged cells is not essential for, neither it enhances interneuronal hα-syn propagation. Data in our model are also consistent with an inverse correlation between neuronal injury and hα-syn spreading. Mechanisms underlying this effect are unclear. It is conceivable that an enhanced stress response within dying cells (e.g., changes in protein degradation) may negatively modulate hα-syn transmission. An alternative and possibly complementary explanation is that neuron-to-neuron α-syn passage involves active mechanisms between relatively healthy cells and is therefore most efficient when neuronal integrity is maintained. Important corollaries to this latter interpretation include the facts that (i) neuronal activity in the form, for example, of synaptic transmission may modulate α-syn propagation [6,40], (ii) specific mechanisms, such as exocytosis, could play a role in protein release and subsequent α-syn transmission [14-16], and (iii) future work aimed at elucidating these mechanisms has the potential to identify new targets for therapeutic intervention against protein spreading in human synucleinopathies.

## Additional files

Additional file 1: Figure S1. Steps involved in the production of the two AAV preps used in this study. The reporter plasmid contained DNA coding for human Synapsin1 (promoter), hα-syn, WPRE (enhancer) and polyA (enhancer). This plasmid was used for transfection of 293 HEK cells. Crude cell lysates were then purified by either (i) two consecutive CsCl gradient centrifugations (AAV prep 1), or (ii) centrifugation through a discontinuous iodixanol gradient followed by heparin affinity chromatography (AAV prep 2). Additional file 2: Figure S2. Delineation of the rat DMnX for stereological cell counting. Every sixth section throughout the entire DMnX was used for stereological counting, yielding 8–9 sections/rat. Representative images of four of these sections at Bregma **(a)** -14.04, **(b)** -13.76, **(c)** -13.68 and **(d)** -13.36 were stained with cresyl violet. The DMnX is delineated in red. Scale bar = 100 μm. Additional file 3: Figure S3. Rats received a single injection of vehicle (control animals, n=3), GFP-AAV prep 1 (n=7) or GFP-AAV prep 2 (n=4) into the vagus nerve. Stereological counts of Nissl-stained DMnX neurons were performed at 12 weeks post treatment on tissue sections from the left (injected side) MO. Values (means ± SEM) represent: [number of neurons in the DMnX ipsilateral to the injection side] / [number of neurons in the DMnX contralateral to the injection side] x 100. * *P* <0.0001 *vs.* the control or prep 2 group (one-way ANOVA followed by Tukey’s post hoc test, F_2,13_ = 22.62).

## Abbreviations

α-syn: Alpha-synuclein
AAV: Recombinant adeno-associated virus
ChAT: Choline acetyltransferase
CTCF: Corrected total cell fluorescence
DMnX: Dorsal motor nucleus of the vagus nerve
GFP: Enhanced green fluorescent protein
hα-syn: Human wild-type alpha-synuclein
MO: Medulla oblongata
PARP: Poly ADP ribose polymerase
PD: Parkinson’s disease
PolyA: Polyadenylation signal sequence
RT-PCR: Reverse transcription polymerase chain reaction
SNc: Substantia nigra pars compacta
TBS: Tris-buffered saline
TBS-T: TBS containing Triton-X-100
WRPE: Woodchuck hepatitis virus post-transcriptional regulatory element
